# Predictors of tooth loss: A machine learning approach

**DOI:** 10.1371/journal.pone.0252873

**Published:** 2021-06-18

**Authors:** Hawazin W. Elani, André F. M. Batista, W. Murray Thomson, Ichiro Kawachi, Alexandre D. P. Chiavegatto Filho

**Affiliations:** 1 Department of Oral Health Policy and Epidemiology, Harvard School of Dental Medicine, Boston, Massachusetts, United States of America; 2 Department of Health Policy and Management, Harvard T. H. Chan School of Public Health, Boston, Massachusetts, United States of America; 3 Department of Epidemiology, University of Sao Paulo Public Health School, Sao Paulo, Brazil; 4 Faculty of Dentistry, The University of Otago, Dunedin, New Zealand; 5 Department of Social and Behavioral Sciences, Harvard T. H. Chan School of Public Health, Boston, Massachusetts, United States of America; Taipei Medical University, TAIWAN

## Abstract

**Introduction:**

Little is understood about the socioeconomic predictors of tooth loss, a condition that can negatively impact individual’s quality of life. The goal of this study is to develop a machine-learning algorithm to predict complete and incremental tooth loss among adults and to compare the predictive performance of these models.

**Methods:**

We used data from the National Health and Nutrition Examination Survey from 2011 to 2014. We developed multiple machine-learning algorithms and assessed their predictive performances by examining the area under the receiver operating characteristic curve (AUC), accuracy, sensitivity, specificity, and positive and negative predictive values.

**Results:**

The extreme gradient boosting trees presented the highest performance in the prediction of edentulism (AUC = 88.7%; 95%CI: 87.1, 90.2), the absence of a functional dentition (AUC = 88.3% 95%CI: 87.3,89.3) and for predicting missing any tooth (AUC = 83.2%; 95%CI, 82.0, 84.4). Although, as expected, age and routine dental care emerged as strong predictors of tooth loss, the machine learning approach identified additional predictors, including socioeconomic conditions. Indeed, the performance of models incorporating socioeconomic characteristics was better at predicting tooth loss than those relying on clinical dental indicators alone.

**Conclusions:**

Future application of machine-learning algorithm, with longitudinal cohorts, for identification of individuals at risk for tooth loss could assist clinicians to prioritize interventions directed toward the prevention of tooth loss.

## Introduction

Tooth loss is considered the “end state” of dental disease [1] and can adversely affect individuals’ general health, quality of life, and well-being [2, 3]. Although its prevalence has declined over the past decade, the aging population means that the risk of tooth loss is expected to rise [4]. Moreover, low-income and marginalized populations still experience a disproportionate share of the burden [5, 6].

Tooth loss can generally be prevented if dental disease is diagnosed and treated at an early stage. Evidence from longitudinal studies suggests that routine dental attenders lose fewer teeth [7]. However, ongoing barriers to access to dental care, including its high cost, limit utilization of dental services, particularly among low-income and minority populations [8]. Adult dental coverage is not an essential health benefit in most public health insurance programs in the United States. Thus, even when able to access dental services, a large proportion of low-income adults have poor oral health due to a lack of routine care, and extraction becomes the most affordable and expedient dental treatment. Identification of individuals at high risk of tooth loss could therefore (a) aid clinicians in implementing early prevention, and (b) inform policies to ensure access to dental care and improve the oral health of vulnerable populations.

While prior research indicates that dental caries remains (by far) the greatest contributor to tooth loss [9], followed by periodontal disease [10], the role of socioeconomic conditions and other health characteristics is less clear. This is primarily because most prior analyses were based on descriptive studies with a limited number of variables [11, 12]. Machine-learning algorithms comprise an approach that utilizes information on a large number of characteristics to identify variables that predict an outcome. This procedure relies on pattern recognition by training the algorithm using “training data” to identify complex patterns to predict outcomes in a separate data “test data” and are therefore better able to model non-linear and high-dimensional characteristics, which is the case of most health data [13, 14]. Machine-learning methods have been recently applied in medicine to provide information to support clinical decisions, such as in predicting survival in cancer patients or survival in intensive care units [14, 15]. However, little is known about developing machine-learning algorithms for the prediction of oral health outcomes [13]. Our objective is to build on that evidence and develop and test multiple machine-learning algorithms to predict complete and incremental tooth loss among adults using socioeconomic and medical condition predictors and to compare the predictive performance of those developed models.

## Methods

### Study population and data sources

**Fig 1:**
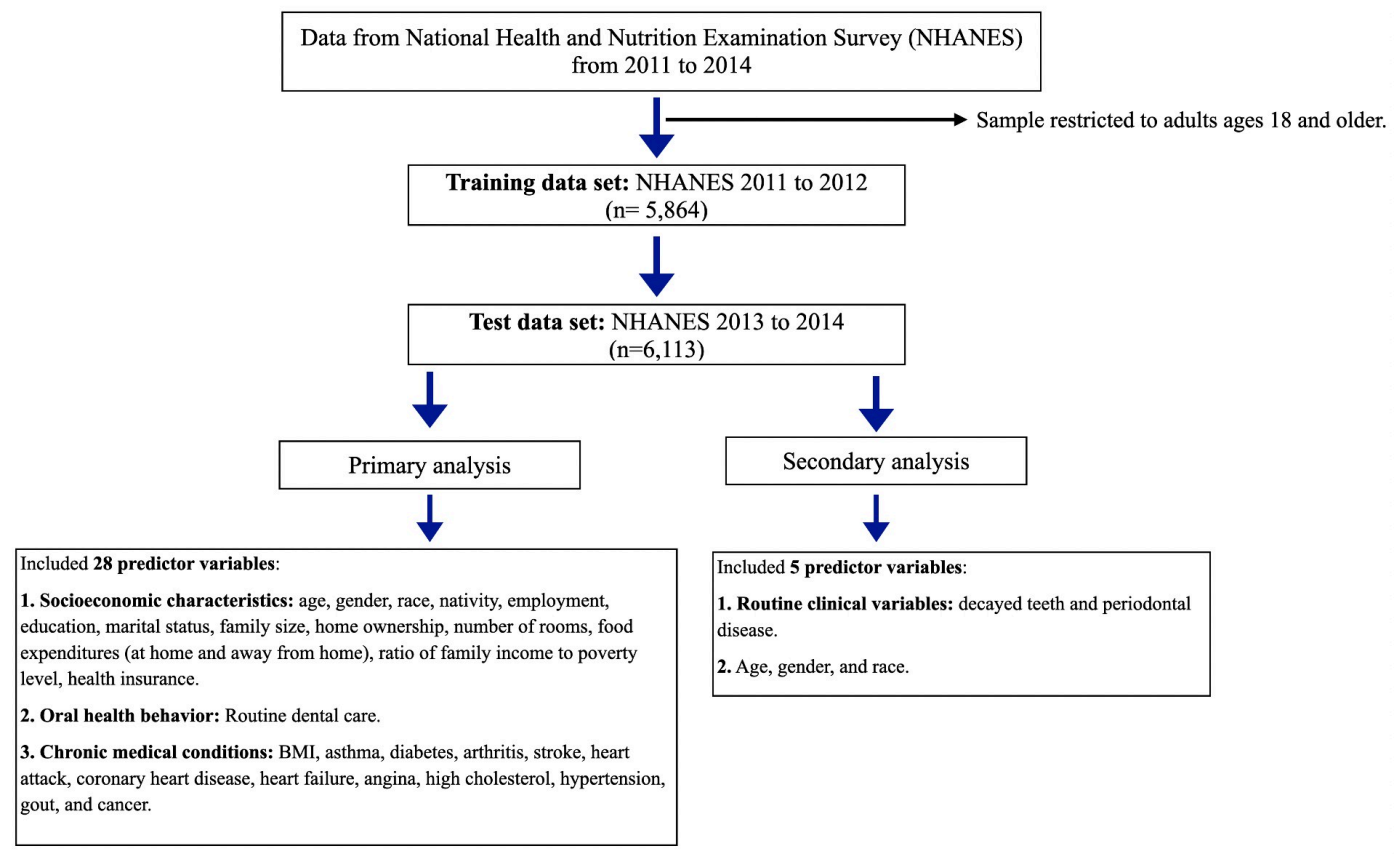
Study flow diagram.

We analyzed data from the National Health and Nutrition Examination Survey (NHANES), conducted by the National Center for Health Statistics [16]. NHANES use stratified multistage probability samples of the civilian non-institutionalized population of the US. NHANES surveys contain information on sociodemographic data, medical conditions, in addition to detailed dental examination. We restricted our sample to adults ages 18 and older. We used data from NHANES cycle 2011 to 2012 (n = 5,864) to develop the predictive models for each outcome “training set”, and we then used cycle 2013 to 2014 (n = 6,113) to test our models’ performance in new unseen data (Fig 1).

### Study variables

Our outcome variables were: (1) edentulism, which is the complete loss of all natural teeth; (2) the presence or absence of a functional dentition, which is defined as having at least 20 teeth [17]; and (3) having one or more missing teeth. All outcomes were dichotomized (yes, no). In our primary analyses, we included a total of 28 socioeconomic characteristics, oral health behavior, and chronic medical conditions as predictors for the machine-learning algorithms. Those variables included age, gender, race, nativity, employment, education, marital status, family size, home ownership, number of rooms, food expenditures (at home and away from home), ratio of family income to poverty level, health insurance, body mass index (BMI), routine dental care, and self-reported diagnoses of asthma, diabetes, arthritis, stroke, heart attack, coronary heart disease, heart failure, angina, high cholesterol, hypertension, gout, and cancer.

In secondary analysis, we ran a model which included only routine clinical variables that clinicians might rely upon to predict future tooth loss in patients, viz., the number of decayed teeth and periodontal disease, in addition to age, gender, and race. Detailed description of predictor variables is shown in S1 Table.

### Statistical analysis

We tested five popular machine-learning algorithms to predict each outcome. These algorithms were logistic regression, random forest (ensemble of multiple decision trees with bootstrap aggregating), light gradient boosting machine and extreme gradient boosting trees (both based on sequential models of decision trees), and artificial neural networks (algorithms inspired by neural structures and trained with back propagation).

We performed one-hot encoding for every categorical variable and standardized continuous variables to avoid oversized effects due to differences in scale. We applied 10-fold cross-validation to tune hyperparameters with Bayesian optimization (hyperopt) for the training set to avoid overfitting, separately for each outcome. For edentulism, due to a small number of positive values (reflecting low prevalence), the training set was resampled with one-side selection. In imbalanced datasets, machine learning algorithms have a tendency of biasing decisions towards the majority class. A common solution to this problem is to undersample the majority class. We applied one-sided selection to the training set, which is an undersampling method that removes examples from the majority class that are noisy and distant from the decision border.

After selecting the combination of hyperparameters with the highest area under the receiver operating characteristic curve (AUC) for each model, the parameters of the final algorithms were defined with the entire 2011–12 NHANES cycle (training set) and their predictive performance tested on the 2013–14 cycle of NHANES (test set). All of the results presented here are from the test set.

To assess the predictive performance of the algorithms, we calculated the AUC, accuracy (ACC), sensitivity, specificity, positive predictive value (PPV), negative predictive value (NPV), and the harmonic mean for sensitivity and specificity for each predictive model. We used 50% threshold for reporting sensitivity, specificity, F1, PPV, NPV. However, in a sensitivity analyses we also tested two other thresholds 25% and 75% (S2 Table).

Furthermore, we computed Shapley values for each predictive model to determine the importance of each variable in predicting our study outcomes. Shapley values are an additive feature importance measure that represent the responsibility of each feature in pushing the model output away from its base value [18].

We used Python (scikit-learn library) [19] and STATA 15.1 software for our analyses [20]. NHANES surveys are approved by National Center for Health Statistics (NCHS) Research Ethics Review Board (ERB) [21]. This study used deidentified data and was determined to be “not-human subjects research” by the institutional review board of the of the Harvard Faculty of Medicine.

## Results

The study included a total of 11,977 adults. There were 736 (5.3%) individuals who were edentulous, 2,663 (18.5%) adults without a functional dentition, and 6,919 (58.3%) adults missing at least one tooth. Nearly half of the sample were women (51.8%) and the majority had more than high school education (63.0%) and were non-Hispanic white (65.7%). The distribution of demographic and health characteristics was relatively similar across the three outcomes (Table 1).

**Table 1:** Demographic characteristics of the study sample: National Health and Nutrition Examination Survey (2011–2014).

	Full sample		Edentulous sample	Having fewer than 21 teeth	Missing any tooth
	N = 11,977		N = 736	N = 2,663	N = 6,919
	n	(%)*	Survey-Weighted Proportions		
**Variable**					
**Sex**					
Male	5,813	(48.2)	48.0	48.1	48.3
Female	6,164	(51.8)	52.0	51.9	51.7
**Age, y ± SD**	46.4	± 17.5	65.7 ± 13.7	61.7 ± 15.0	52.9 ± 16.8
**Education** ^a^					
Less than high school degree	2,578	(16.0)	40.1	31.0	20.7
High school graduate	2,472	(21.0)	29.1	31.2	24.6
Some college/college graduate	6,267	(63.0)	30.9	37.7	54.7
**Race/ethnicity**					
Non-Hispanic White	4,679	(65.7)	74.1	64.6	64.8
Non-Hispanic Black	2,809	(11.6)	12.5	17.0	13.3
Hispanic	2,594	(14.7)	6.3	11.5	14.4
Other	1,895	(7.9)	7.1	6.9	7.4
**Nativity**					
US-born	8,463	(82.4)	89.2	84.8	82.2
Foreign born	3,505	(17.6)	10.8	15.2	17.8
**Family income, % of FPL**					
<100	2,770	(17.4)	29.8	24.7	18.8
100–200	2,851	(21.5)	35.1	32.3	24.7
>200	5,339	(61.2)	35.1	43.0	56.5
**Health insurance**					
Insured	9,279	(81.1)	89.7	83.0	80.2
Uninsured	2,680	(18.9)	10.3	17.0	19.8
**Routine dental care**					
Yes	4,379	(44.7)	35.4	19.9	37.3
No	7,388	(55.3)	96.5	80.1	62.7
**BMI**					
≤24.99	3,695	(31.1)	29.6	25.9	27.5
25.0–29.99	3,595	(32.9)	33.4	31.7	33.7
≥30	4,082	(36.0)	37.0	42.3	38.8
**Medical conditions**					
Asthma	1,815	(15.5)	17.3	15.6	14.7
Arthritis	2,873	(24.9)	52.2	45.8	31.9
Diabetes	1,432	(9.4)	23.5	20.6	13.4
Hypertension	4,180	(32.5)	60.5	56.0	41.6
High cholesterol levels	3,846	(33.7)	52.8	49.5	39.5
Stroke	431	(2.9)	13.9	9.0	4.2
Heart attack	433	(3.3)	14.2	9.7	5.2

***Note***.

^a^ Education is based on individuals ages 20 years and older. Wisdom teeth were excluded, and all analyses were based on a maximum of 28 teeth.

* Survey-Weighted Proportion. SD is standard deviation.

The performance of the machine-learning algorithms on the test data for each study outcome, for the primary analyses (without dental clinical variables), is summarized in Table 2. For edentulism; all machine-learning models demonstrated high performance with high AUC (>86.5%). The ACC ranged between 82.2% and 84.3%, indicating good accuracy. The sensitivity ranged between 71.9% and 78.5%, while specificity was high (>82.5%) for all models. In predicting the lack of a functional dentition, all models had high performance, with high AUC and ACC (>87.0% and >81.0% respectively). The specificity was greater than 84.0% and the sensitivity ranged between 48.4% and 74.1%. For predicting one or more missing teeth, the AUC were greater than 81.0% and ACC more than 73.0%. The sensitivity for all models was high (>85.0%), and the specificity ranged between 29.6% and 59.7%. The performance of the machine-learning algorithms on the train data for each study outcome is presented in (S3 Table).

**Table 2:** Performance of the machine-learning algorithms on the test data for each study outcome.

	AUC	ACC	Sensitivity	Specificity	F1	PPV	NPV	Harmonic
	(95% CI)							Mean
**Edentulism**								
Classifier								
Extreme gradient boosting trees	88.7 (87.1, 90.2)	83.8	74.3	84.5	39.4	26.8	97.7	79.0
Random forests	88.5 (86.9, 90.0)	84.3	73.7	85.1	40.1	27.5	97.7	78.9
Neural networks	87.7 (86.0, 89.3)	82.2	78.5	82.5	38.6	25.6	98.1	80.4
Light gradient boosting machine	88.4 (86.7, 89.9)	82.7	76.4	83.1	38.5	25.7	97.9	79.6
Logistic regression	86.5 (84.7, 88.3)	83.7	71.9	84.6	38.5	26.3	97.5	77.7
**Having fewer than 21 teeth**								
Classifier								
Extreme gradient boosting trees	88.3 (87.3, 89.3)	81.5	74.1	84.2	68.1	62.9	90.0	78.8
Random forests	87.6 (86.5, 88.6)	81.7	48.4	93.7	58.4	73.5	83.4	63.8
Neural networks	88.1 (87.0, 89.1)	82.6	56.4	92.0	63.2	71.9	85.4	69.9
Light gradient boosting machine	87.7 (86.7, 88.7)	82.5	58.0	91.3	63.7	70.7	85.7	70.9
Logistic regression	87.2 (86.2, 88.3)	81.9	53.9	92.1	61.3	71.0	84.7	68.0
**Missing any tooth**								
Classifier								
Extreme gradient boosting trees	83.2 (82.0, 84.4)	74.0	95.9	29.6	83.2	73.4	77.8	45.2
Random forests	82.7 (81.4, 83.8)	77.0	89.5	55.6	83.6	80.0	68.6	68.5
Neural networks	83.1 (81.9, 84.3)	77.2	85.8	59.7	83.5	81.2	67.5	70.4
Light gradient boosting machine	81.9 (80.6, 83.0)	73.9	93.7	33.8	82.8	74.2	72.6	49.6
Logistic regression	83.1 (81.9, 84.3)	76.9	85.6	59.4	83.3	81.1	67.0	70.1

***Note*.** Test data: National Health and Nutrition Examination Survey (NHANES 2013–2014). AUC = area under the receiver operating characteristic curve; ACC = accuracy; PPV = positive predictive value; NPV = negative predictive value; F1 = F1 score; Harmonic mean = between sensitivity and specificity.

**Fig 2:**
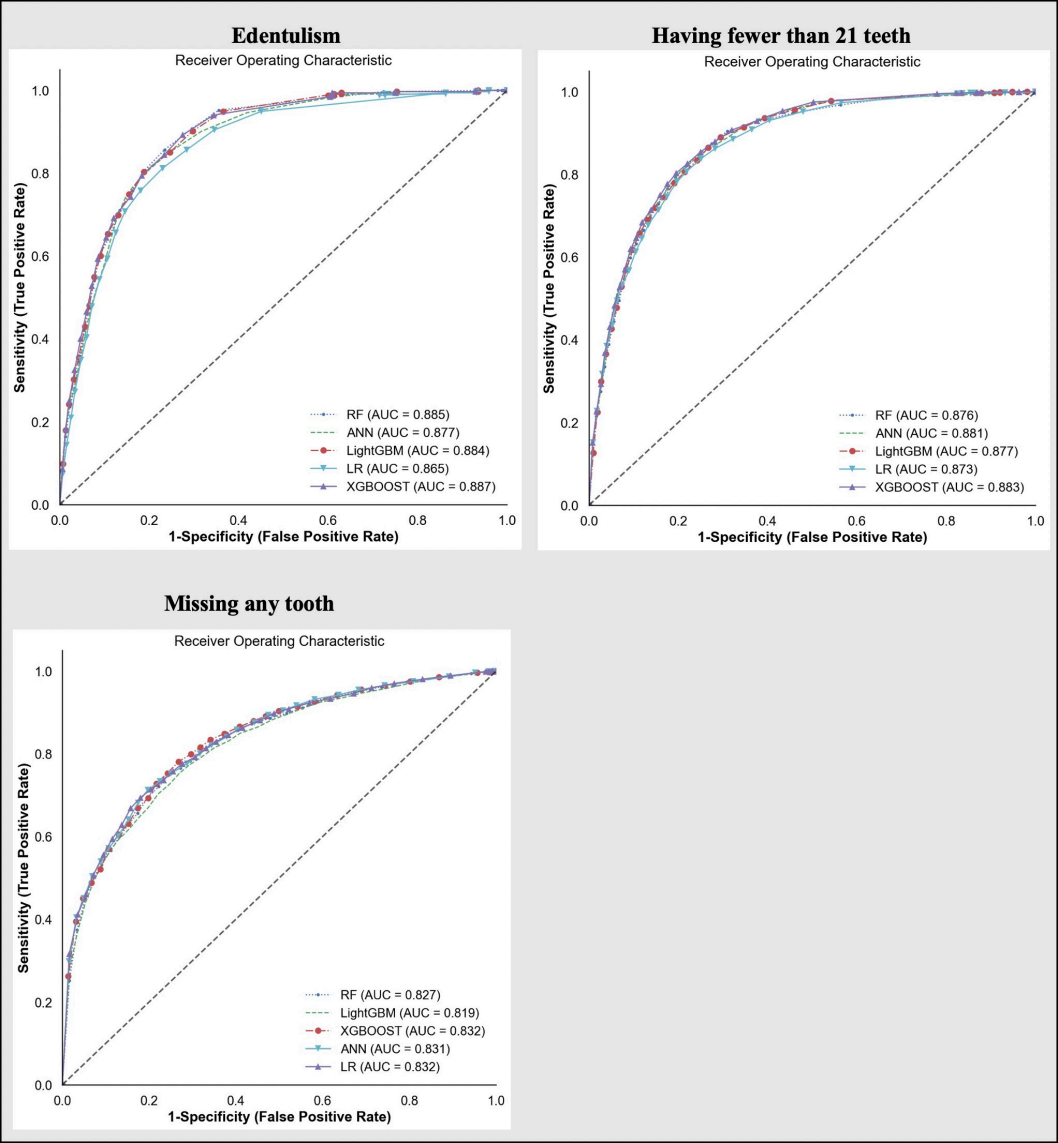
Receiver-operating characteristics curves for the five analyzed predictive models for each outcome.

We compared the AUC curves from all machine-leaning algorithms by outcome (Fig 2). Generally, all models were very similar demonstrating high AUC (>81.5%). Considering all performance parameters (Table 2), the extreme gradient boosting trees had the highest performance in predicting all outcomes. The AUC for extreme gradient boosting trees in predicting edentulism was 88.7% (95%CI: 87.1, 90.2); for not having a functional dentition it was 88.3% (95%CI: 87.3, 89.3), and for predicting one or more teeth missing it was 83.2%; 95%CI: 82.0, 84.4). The final hyperparameters for all predictive models are presented in S4 Table.

**Fig 3:**
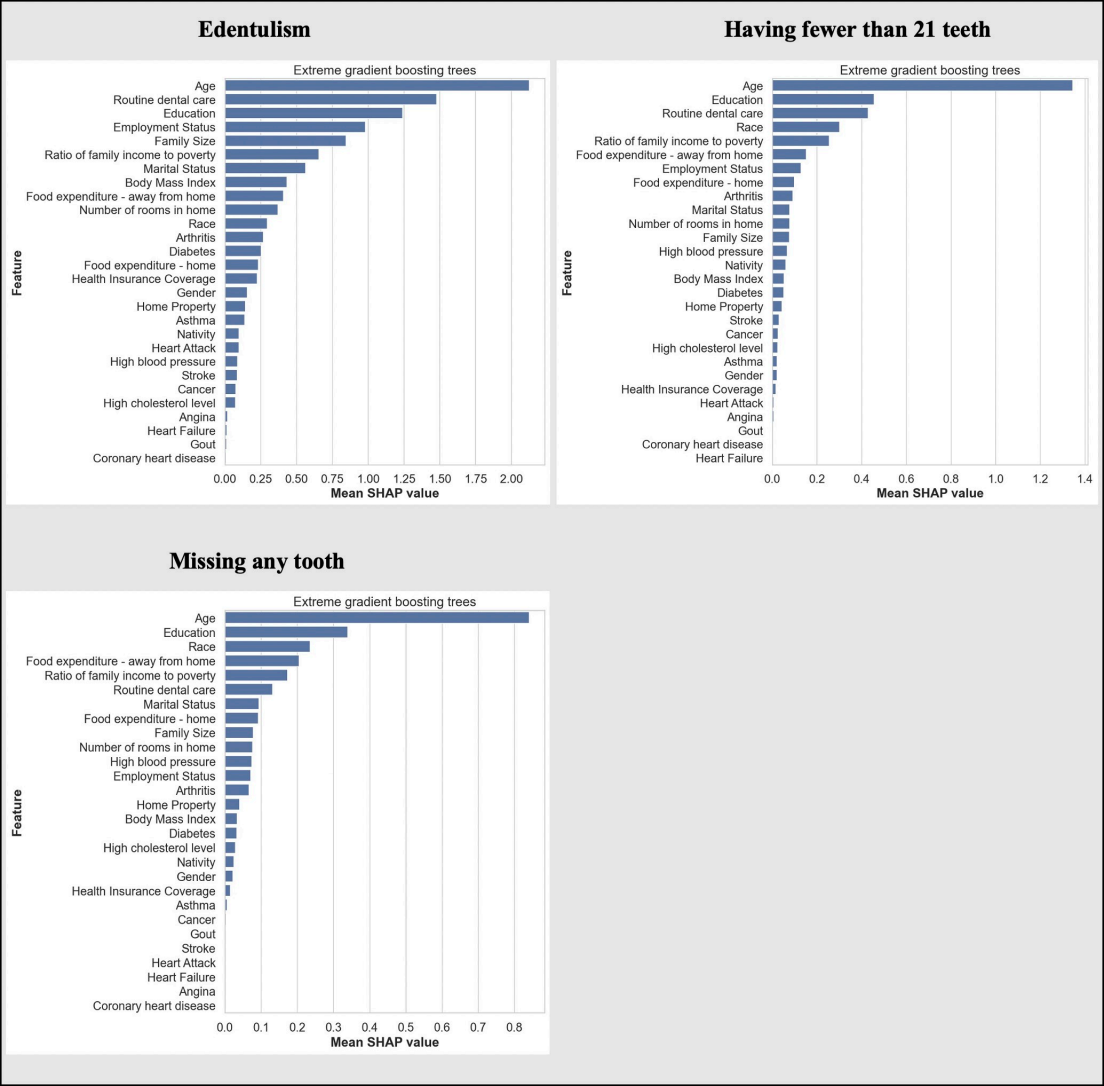
Variable importance plot in the extreme gradient boosting trees models for each outcome.

The most important predictive variables, from the best performing classifiers for each outcome, were nearly similar for all outcomes (Fig 3). Age, education, routine dental care, employment, ratio of family income to poverty level, race, and home ownership were strong predictors of tooth loss. While less significant, medical conditions such as arthritis, diabetes, high cholesterol, hypertension, and cardiovascular diseases were also among the list of predictors. The variable rankings are robust throughout the different machine learning models and different outcomes, with age, education and race emerging as the top predictors for most models (S1–S3 Figs).

Results from our secondary analyses—using only routine clinical variables to predict tooth loss—are presented in Table 3. The performance of these machine-learning algorithms showed that for edentulism, the logistic regression had the highest predictive performance (AUC = 84.6%; 95% CI: 83.0, 86.1). The extreme gradient boosting trees demonstrated the highest performance in predicting the absence of a functional dentition (AUC = 80.4%; 95% CI: 78.9, 81.7) and for predicting one or more teeth missing (AUC = 79.8%; 95% CI: 78.2, 81.2). In each case, the algorithms using only clinical variables (number of decayed teeth, periodontal disease) performed worse than the algorithms excluding the same variables, but incorporating socioeconomic factors.

**Table 3:** Performance of the machine-learning algorithms on the test data for each study outcome when including clinical dental predictors^a^.

	AUC	ACC	Sensitivity	Specificity	F1	PPV	NPV	Harmonic
	(95% CI)							Mean
**Edentulism**								
Classifier								
Extreme gradient boosting trees	83.9 (82.1, 85.5)	83.9	52.1	86.9	35.9	27.4	95.0	65.1
Random forests	78.0 (75.3, 80.6)	80.7	61.9	82.5	35.7	25.1	95.8	70.7
Neural networks	83.7 (82.0, 85.3)	77.1	73.4	77.4	35.7	23.6	96.8	75.3
Light gradient boosting machine	83.0 (81.2, 84.8)	81.5	61.3	83.4	36.5	25.9	95.8	70.6
Logistic regression	84.6 (83.0, 86.1)	76.6	77.7	76.5	36.4	23.8	97.3	77.1
**Having fewer than 21 teeth**								
Classifier								
Extreme gradient boosting trees	80.4 (78.9, 81.7)	75.7	45.0	89.6	53.6	66.3	78.2	59.9
Random forests	80.0 (78.5, 81.4)	75.1	42.3	90.0	51.4	65.7	77.4	57.5
Neural networks	80.3 (78.9,81.7)	75.5	50.4	87.0	56.3	63.8	79.4	63.8
Light gradient boosting machine	79.1 (77.7, 80.5)	71.3	72.3	70.8	61.1	53.0	84.9	71.5
Logistic regression	79.3 (77.9, 80.7)	74.9	47.3	87.4	54.1	63.0	78.5	61.3
**Missing any tooth**								
Classifier								
Extreme gradient boosting trees	79.8 (78.2, 81.2)	76.9	93.1	29.6	85.7	79.4	59.6	44.9
Random forests	79.8 (78.2, 81.2)	75.6	98.8	8.0	85.8	75.8	69.5	14.8
Neural networks	79.4 (77.8, 80.8)	76.7	89.3	40.1	85.1	81.3	56.3	55.3
Light gradient boosting machine	78.3 (76.7, 79.8)	75.7	98.6	8.7	85.8	75.9	68.7	15.9
Logistic regression	79.5 (78.0, 81.0)	76.8	91.3	34.6	85.5	80.3	57.8	50.2

***Note*.** Test data: National Health and Nutrition Examination Survey (NHANES 2013–2014). AUC = area Under the receiver operating characteristic curve; ACC = accuracy; PPV = positive predictive value; NPV = negative predictive value; F1 = F1 score; Harmonic mean = between sensitivity and specificity.

^a^ Predictor variables included are the number of decayed teeth, periodontal disease, age, gender, and race.

## Discussion

To the best of our knowledge, this is the first use of machine-learning algorithms to predict complete and incremental tooth loss based on socioeconomic and medical health characteristics [22]. In this study, we used national data to develop and test the performance of five machine-learning algorithms and to identify predictors of complete and incremental tooth loss. We assessed the predictive performances of our models by examining several parameters, including area under the receiver operating characteristic curve, accuracy, sensitivity, specificity, positive and negative predictive values. Overall, all machine-learning models demonstrated high predictive performance with high discrimination, achieving AUC greater than 82.0%. We found that the extreme gradient boosting trees model had the highest performance in predicting edentulism, the absence of a functional dentition, and missing any tooth.

Tooth loss is an important oral health indicator. Depending on its severity, it can significantly impact the ability to chew, speak, socialize, and overall general health [2, 3, 23]. While previous research has identified the determinants of tooth loss, most of this literature examined only edentulism and did not examine incremental tooth loss, which is a far more prevalent oral state [11]. Moreover, those studies were mostly based on cross-sectional data or examined a few variables using classical statistical modeling rather than predictive modeling [11, 12, 24]. We found that machine-learning models performed better than conventional statistical methods (logistic regression) for predicting edentulism and the absence of a functional dentition. Similarly, Krois et al recently developed and evaluated the performance of multiple predictive models for tooth loss in patients with periodontal disease. Their study demonstrated the utility of applying machine-leaning framework for predicting tooth loss mainly from periodontal tooth-level predictors [13].

In this study, we assessed the utility of machine-learning algorithms for predicting complete and incremental tooth loss, and we demonstrated a high predictive performance of those models. We did not include clinical dental variables in the primary analyses since they are generally highly correlated with tooth loss [9, 13, 25, 26]. Indicators such as decayed teeth and poor periodontal condition have been documented as strong determinants for tooth loss; when we included them, our prediction models were nearly perfect. Instead, we used a comprehensive list of socioeconomic characteristics, self-reported dental care, and medical condition variables. Our approach aimed to develop predictive models using variables that do not require dental examination so that non-dental clinicians could readily identify this high-risk population. However, in our secondary analyses, we assessed the predictive performance of the machine-learning algorithms for predicting our outcomes using a limited number of dental clinical predictors and demographic variables. Our findings suggest that the machine-learning algorithms models using socioeconomic characteristics, self-reported dental care, and medical condition variables performed better at predicting tooth loss than relying on clinical dental indicators alone. Knowing the patient’s education level, employment status, and income is just as relevant for predicting tooth loss as assessing their clinical dental status. Our findings echo the advice of Bernardino Ramazzini (1633–1714), widely considered to be the father of occupational medicine, who admonished clinicians to always ask about their patients’ occupation when taking down their medical histories [27].

Our findings are consistent with those of previous studies to have identified age and socioeconomic conditions as risk factors for tooth loss [11, 12, 28]. Aging populations have accumulated oral and non-communicable health conditions, and so remain susceptible to ongoing tooth loss. We also found education to be another strong predictor of tooth loss. Education is a marker of socioeconomic position and a key determinant of life chances, opportunities, beliefs, and values; it therefore plays an important role in enabling access to (and affordability of) dental services [29, 30]. Routine dental care also emerged as a strong predictor of tooth loss. This finding provides support for the association between regular preventive dental visits and better oral health [7]. Our findings also provide insights into the role of pre-existing medical conditions as determinants of tooth loss. We found that medical conditions—such as arthritis, diabetes, high cholesterol, hypertension and cardiovascular diseases—are among the predictors of tooth loss. Clinicians could use this information to screen patients at high risk for tooth loss and coordinate their referral and dental care.

Even though the association between socioeconomic status (SES) and tooth loss has been documented previously, we believe our study makes a novel contribution by providing a direct comparison with the predictive performance of widely accepted clinical indicators. We believe our approach builds on prior knowledge by quantitatively demonstrating—via machine-learning algorithm—that a set of socioeconomic variables perform better than clinical dental indicators in predicting tooth loss. Again, we cannot establish causality (which would require longitudinal data), but it draws attention to the potential utility of incorporating SES among a set of variables that clinicians ought to consider in their practice. Future studies need to evaluate the application of these algorithms in clinical settings and explore their use in the identification of populations at risk for other dental outcomes.

Previous studies in other areas of clinical practice have pointed out the potential utility of incorporating socioeconomic information in prediction algorithms. For example, the Framingham Risk calculator–one of the most widely used algorithms to predict future risk of cardiovascular disease–currently does not incorporate socioeconomic variables. Studies have suggested that this omission results in the systematic under-treatment of low-SES patients with hyperlipidemia, because the Framingham Risk score under-estimates the risk of CVD in low SES patients, and clinicians’ treatment decisions (such as when to start statin therapy) are often based on stratifying patients using the same algorithms [31, 32]. Although we cannot cite an example of a comparable example in dentistry, our study has potential implications for any future attempts to develop prediction algorithms to guide decision making.

A very limited number of studies have utilized machine-learning approaches, mostly developing a single algorithm model or using clinical dental variables, for predicting oral health outcomes [13, 33, 34]. Our study builds on prior findings and demonstrates the feasibility of using this approach in predicting tooth loss. Future studies could utilize machine-learning for predicting populations at risk for other dental outcomes.

We used cross-sectional data in this study. Although we used separate cycles from NHANES data to develop and to test the predictive models, the principal threat to causal inference in cross-sectional data is reverse causation. Studies have suggested that poor dentition is, indeed, a predictor of low SES, e.g. because individuals with “poor oral health” are less likely to be selected at job interviews [35, 36]. However this type of reverse causation is less likely for education, since missing teeth is less likely to affect educational attainment. The evidence is stronger for job hiring, promotion, and income. Future studies with longitudinal cohorts with a larger time separation between training and test datasets are needed to test the external validity of our models and ensure robustness of the models’ performance with time. In addition, we excluded variables that had 20% or more missing data from the primary analyses which may have limited the number of variables we were able to test such as other oral heath behaviors (brushing and flossing) and lifestyle factors (smoking, drinking, and exercise). Nonetheless, we were able to develop machine-learning algorithms with high predictive performances for all outcomes. Additionally, we conducted a sensitivity analyses by using multivariate imputation by chained equations (MICE) to impute missing data so that all individuals are included for every model, even if they have missing values and the results were largely similar to our main analysis (S5 Table). Finally, although the performance of models predicting edentulism was high (AUC >86%), the prevalence of edentulism was low in our sample (thus, a “rare event”), and this may have affected the performance of those models.

### Conclusion

In this analysis we developed and tested the performance of five machine-learning algorithms for predicting complete and incremental tooth loss. Our findings support the application of machine-learning algorithms to predict tooth loss using socioeconomic and medical health characteristics. However, future studies will need to validate our models using longitudinal data to aid health policy as well as clinicians in identifying individuals at high risk of tooth loss so that early interventions can be directed at those most at risk. In addition, the application of machine-learning methods can be used to identify predictors of other dental conditions.

## Supporting information

S1 Fig Variable importance plot for different models for edentulism. (PDF)

S2 Fig Variable importance plot for different models for having fewer than 21 teeth. (PDF)

S3 Fig Variable importance plot for different models for missing any tooth. (PDF)

S1 Table Description of predictor variables included in the analyses. (PDF)

S2 Table Performance of the machine-learning algorithms for each study outcome testing multiple metrics thresholds. (PDF)

S3 Table Performance of the machine-learning algorithms on the training data for each study outcome. (PDF)

S4 Table Final hyperparameters for all predictive models for study outcomes. (PDF)

S5 Table Performance of the machine-learning algorithms on the test data for each study outcome with imputed data. (PDF)
