# Supplementary figures and images for: Predictors of tooth loss: A machine learning approach
Source: PLoS One. 2021 Jun 18;16(6):e0252873. doi: 10.1371/journal.pone.0252873 (PMC8213149; doi:10.1371/journal.pone.0252873)

**S1 Fig.** Variable Importance Plot for Different Models for Edentulism.

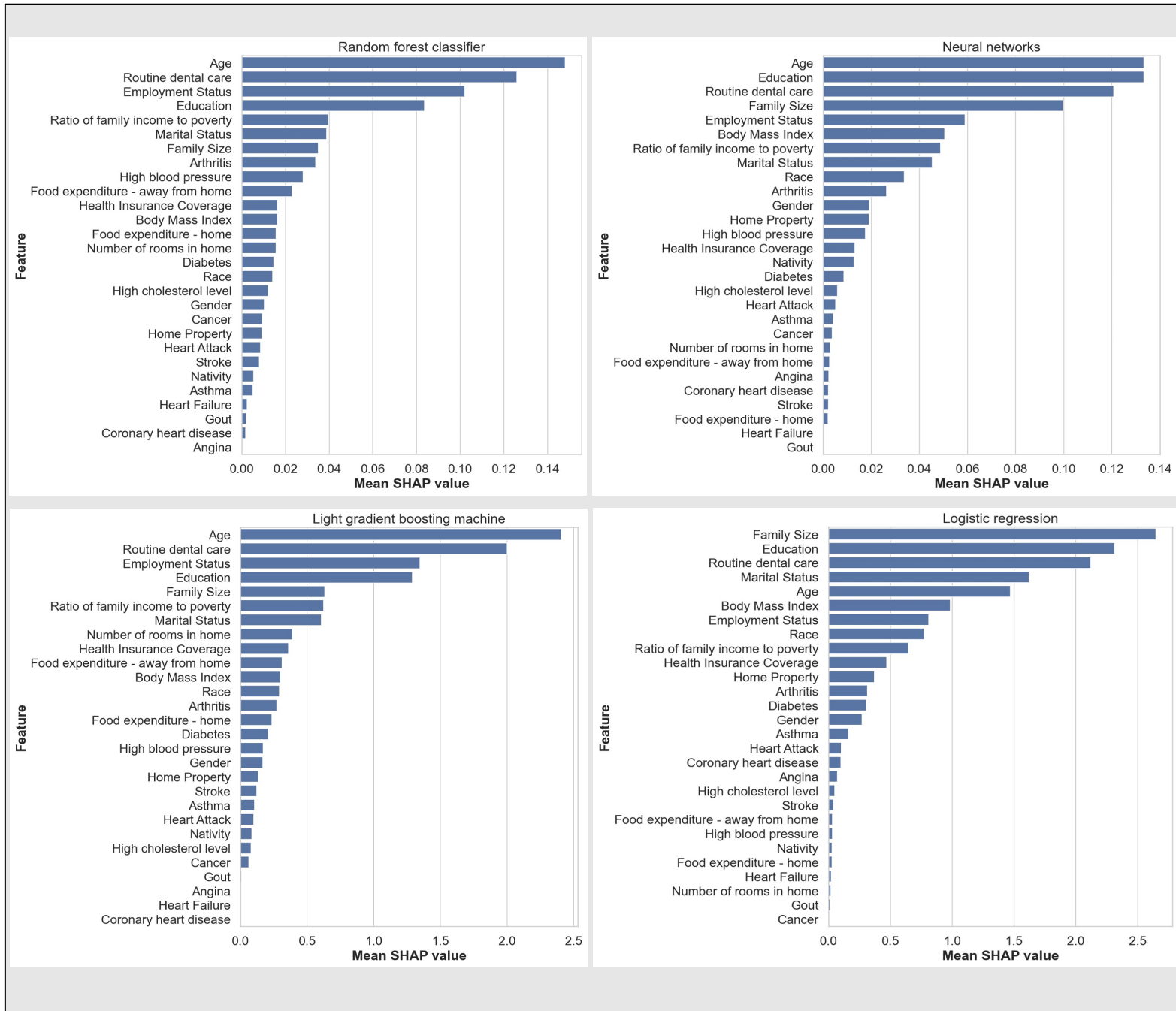

Supplement: S1 Fig — (PDF) [file pone.0252873.s001.pdf]

**S2 Fig.** Variable Importance Plot for Different Models for Having Fewer Than 21 Teeth.

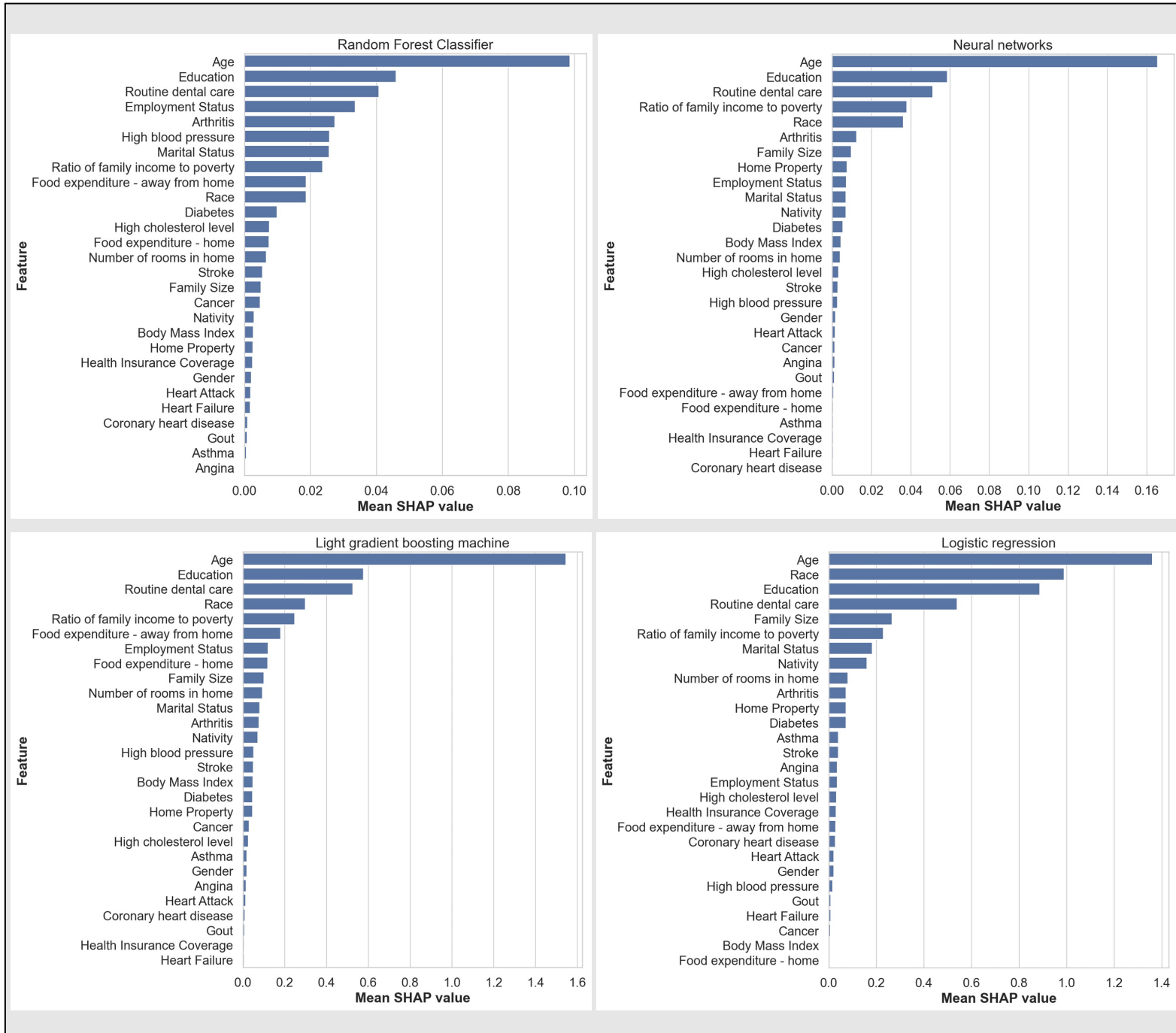

Supplement: S2 Fig — (PDF) [file pone.0252873.s002.pdf]

**S3 Fig.** Variable Importance Plot for Different Models for Missing Any Tooth.

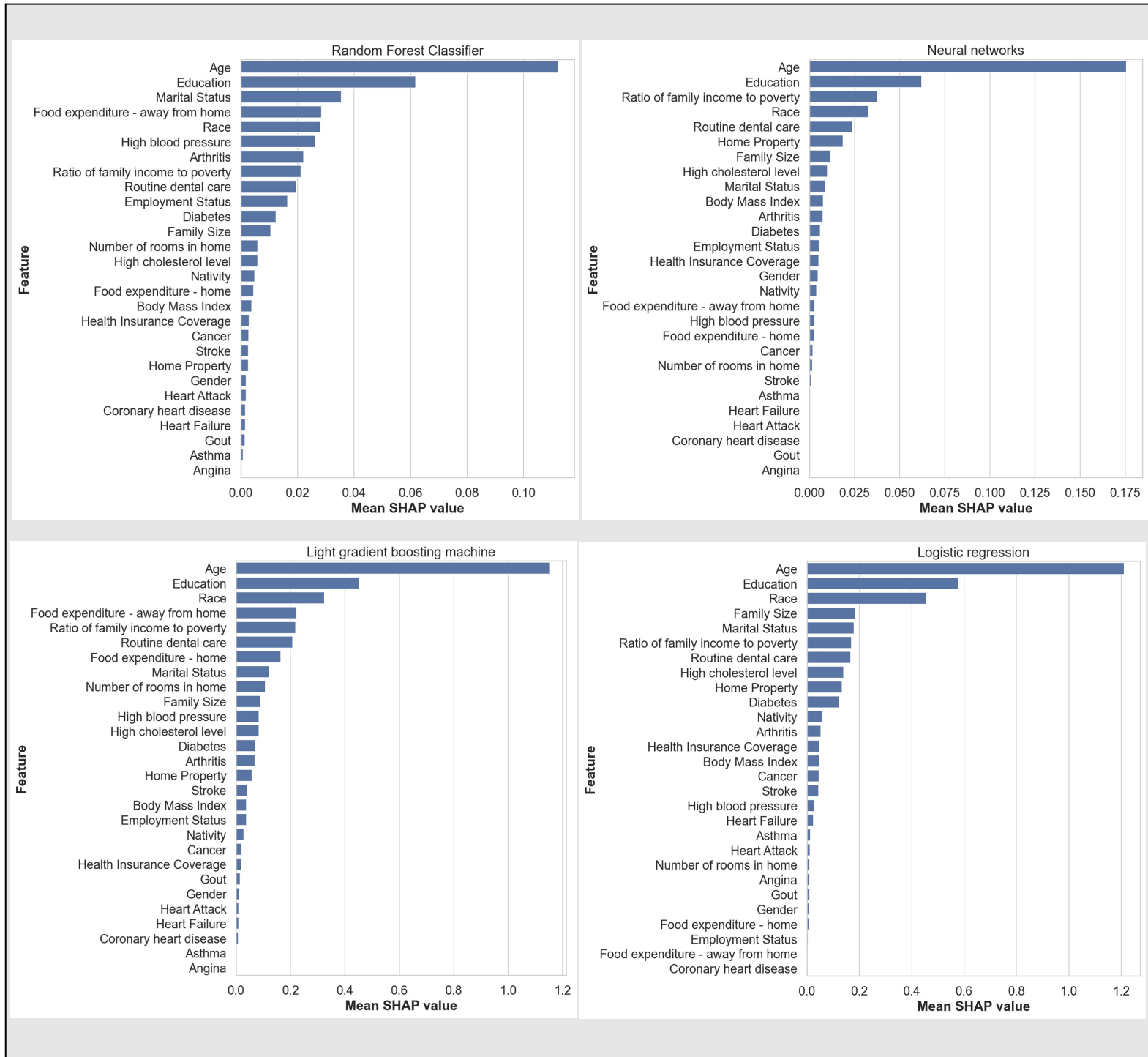

Supplement: S3 Fig — (PDF) [file pone.0252873.s003.pdf]
