# Supplementary material for: Predictors of tooth loss: A machine learning approach
Source: PLoS One. 2021 Jun 18;16(6):e0252873. doi: 10.1371/journal.pone.0252873 (PMC8213149; doi:10.1371/journal.pone.0252873)
Supplement: S1 Table — (PDF) [file pone.0252873.s004.pdf]

S1 Table: Description of Predictor Variables Included in the Analyses.

Variable	Measure
**Socioeconomic variables**	
Age	Age in years at screening (18 to 80 y)
Gender	Male, Female
Race	Non-Hispanic White, Non-Hispanic Black, Mexican American, Other Hispanic, Other race
Nativity	US, Other
Employment	Yes, No
Education	Less than 9th grade, 9-11th grade, HS grad/GED or equivalent, some college or AA degree, or College grad or above
Marital status	Married, Widowed, Divorced, Separated, Never married, or Living with partner
Family size	Total number of people in the Family (1 to 7)
Home ownership	Owned/bought, Rented, or Other arrangement
Number of rooms	1 to 15 rooms
Food expenditures-at home	Money spent at supermarket or grocery store
Food expenditures-away from home	Money spent on eating out, carry out, or delivered food
Ratio of family income to poverty level	0 to 5
Health insurance	Covered by health insurance (yes, no)
BMI	Body Mass Index
**Dental variables**	
Routine dental care	Visited the dentist in the past year for check-up, examination, or cleaning
Decayed teeth	Number of decayed teeth based on clinical examination.
Periodontal disease	Having 2 or more sites with clinical attachment loss of greater than 5 mm and one or more site with probing depth greater than 4 mm.
**Medical conditions**	
Asthma	Ever been told you have asthma (yes, no)
Arthritis	Doctor ever said you had arthritis (yes, no)
Diabetes	Doctor told you have diabetes (yes, no, borderline)
Stroke	Ever told you had a stroke (yes, no)
Heart attack	Ever told you had heart attack (yes, no)
Coronary heart disease	Ever told you had coronary heart disease (yes, no)
Heart failure	Ever told had congestive heart failure (yes, no)
Angina	Ever told you had angina/angina pectoris (yes, no)
High cholesterol	Doctor told you - high cholesterol level (yes, no)
Hypertension	Ever told you had high blood pressure (yes, no)
Gout	Doctor ever told you that you had gout? (yes, no)
