# Supplementary material for: Predictors of tooth loss: A machine learning approach
Source: PLoS One. 2021 Jun 18;16(6):e0252873. doi: 10.1371/journal.pone.0252873 (PMC8213149; doi:10.1371/journal.pone.0252873)
Supplement: S2 Table — (PDF) [file pone.0252873.s005.pdf]

S2 Table: Performance of the Machine-learning Algorithms for Each Study Outcome Testing Multiple Metrics Thresholds on the Training Data.

	**25% cut-off**						**75% cut-off**					
<u>Algorithm</u>	**ACC**	**Sensitivity**	**Specificity**	**F1**	**PPV**	**NPV**	**ACC**	**Sensitivity**	**Specificity**	**F1**	**PPV**	**NPV**
**Edentulism**												
Extreme gradient boosting trees	79.9	82.4	79.7	36.8	23.7	98.3	86.7	68.1	88.1	42.0	30.4	97.3
Random forests	74.9	88.7	73.8	33.4	20.6	98.8	89.8	52.8	92.6	42.3	35.3	96.3
Neural networks	77.9	83.3	77.5	34.9	22.1	98.4	85.7	69.6	86.9	40.9	28.9	97.4
Light gradient boosting machine	79.9	82.7	79.7	36.9	23.8	98.4	85.4	70.1	86.6	40.6	28.6	97.4
Logistic regression	78.9	79.4	78.8	34.8	22.3	98.0	86.4	63.0	88.2	39.8	29.1	96.9
**Having fewer than 21 teeth**												
Extreme gradient boosting trees	74.8	89.9	69.3	65.4	51.4	95.0	82.3	51.2	93.5	60.6	74.0	84.2
Random forests	78.3	83.8	76.3	67.2	56.1	92.9	74.8	6.0	99.7	11.2	86.2	74.6
Neural networks	80.1	79.2	80.5	67.9	59.4	91.5	78.0	22.2	98.2	34.9	81.3	77.7
Light gradient boosting machine	80.0	77.1	81.1	67.2	59.5	90.7	79.2	30.1	96.9	43.5	78.1	79.3
Logistic regression	79.9	77.6	80.7	67.2	59.2	90.9	79.0	26.8	97.9	40.4	81.9	78.7
**Missing any tooth**												
Extreme gradient boosting trees	67.5	99.7	2.2	80.4	67.4	79.1	77.4	82.6	66.9	83.0	83.5	65.4
Random forests	69.1	99.2	7.8	81.1	68.6	83.0	67.7	57.5	88.4	70.5	90.9	50.6
Neural networks	68.4	99.4	5.6	80.8	68.1	82.1	69.6	61.3	86.3	73.0	90.1	52.4
Light gradient boosting machine	68.6	99.2	6.3	80.9	68.3	80.3	75.6	80.6	65.6	81.6	82.6	62.4
Logistic regression	72.7	96.9	23.5	82.6	72.0	78.7	71.0	63.8	85.7	74.7	90.1	53.8
