# Supplementary material for: Predictors of tooth loss: A machine learning approach
Source: PLoS One. 2021 Jun 18;16(6):e0252873. doi: 10.1371/journal.pone.0252873 (PMC8213149; doi:10.1371/journal.pone.0252873)
Supplement: S3 Table — (PDF) [file pone.0252873.s006.pdf]

S3 Table: Performance of the Machine-learning Algorithms on the Training Data for Each Study Outcome.

	AUC (95% CI)	ACC	Sensitivity	Specificity	F1	PPV	NPV	Harmonic Mean
**Edentulism**								
Classifier								
Extreme gradient boosting trees	99.7 (99.6, 99.7)	97.6	98.5	96.4	97.9	97.3	98.0	97.4
Random forests	99.8 (99.8, 99.9)	99.7	99.8	99.6	99.8	99.7	99.7	99.6
Neural networks	99.8 (99.6, 99.8)	98.1	98.8	97.2	98.4	97.9	98.3	97.9
Light gradient boosting machine	98.6 (98.3, 98.8)	95.5	97.4	92.9	96.1	94.8	96.4	95.0
Logistic regression	99.5 (99.4, 99.5)	97.1	97.7	96.2	97.5	97.2	96.9	96.9
**Having fewer than 21 teeth**								
Classifier								
Extreme gradient boosting trees	96.8 (96.3, 97.2)	89.6	93.3	88.3	82.0	73.1	97.5	90.7
Random forests	95.9 (95.3, 96.3)	88.9	65.4	96.9	74.9	87.7	89.1	78.0
Neural networks	90.2 (89.2, 91.1)	85.1	61.6	93.0	67.7	75.1	87.7	74.1
Light gradient boosting machine	97.6 (97.2, 97.9)	92.9	81.1	96.9	85.3	90.0	93.8	88.2
Logistic regression	89.4 (88.3, 90.3)	84.6	61.0	92.6	66.8	73.8	87.5	73.5
**Missing any tooth**								
Classifier								
Extreme gradient boosting trees	90.1 (81.9, 91.0)	78.2	97.9	38.8	85.7	76.2	90.5	55.5
Random forests	95.3 (94.7, 95.8)	87.2	93.8	74.1	90.7	87.9	85.7	82.7
Neural networks	85.6 (88.4, 86.6)	78.8	87.4	61.6	84.6	82.0	71.0	72.2
Light gradient boosting machine	95.2 (94.5, 95.7)	83.6	99.6	51.8	89.0	80.5	98.4	68.1
Logistic regression	84.6 (83.3, 85.7)	78.1	86.0	62.3	83.9	82.0	69.0	72.2

Note. Training data: National Health and Nutrition Examination Survey (NHANES 2011-2012). AUC= area Under the receiver operating characteristic curve; ACC= accuracy; PPV= positive predictive value; NPV= negative predictive value; F1=F1 score; Harmonic mean= between sensitivity and specificity.
