# Supplementary material for: Predictors of tooth loss: A machine learning approach
Source: PLoS One. 2021 Jun 18;16(6):e0252873. doi: 10.1371/journal.pone.0252873 (PMC8213149; doi:10.1371/journal.pone.0252873)
Supplement: S4 Table — (PDF) [file pone.0252873.s007.pdf]

S4 Table: Final Hyperparameters for All Predictive Models for Study Outcomes.

	Edentulism	Having fewer than 21 teeth	Missing any tooth
**XGBClassifier**			
colsample_bylevel	1	1	1
gamma	0.6500	0.9000	0.7000
learning_rate	0.0350	0.0050	0.0050
max_depth	142	142	1067
min_child_weight	10000	60000	40,000
n_estimators	671	1067	1200
reg_alpha	0.5000	0.7500	0.5000
scale_pos_weight	3	3	3
subsample	0.6000	0.5000	0.2000
**RandomForestClassifier**			
bootstrap	FALSE	FALSE	FALSE
class_weight	(0: 1, 1: 4.5)	None	None
criterion	entropy	entropy	entropy
max_depth	142	671	935
max_features	sqrt	sqrt	sqrt
min_samples_leaf	2	7	2
min_samples_split	2	26	31
n_estimators	538	142	935
**LGBMClassifier**			
boosting_type	gbdt	gbdt	gbdt
colsample_bytree	0.6046	0.8502	0.6328
learning_rate	0.1000	0.1000	0.1000
min_child_samples	20	20	20
n_estimators	100	100	100
num_leaves	147	30	30
reg_alpha	0.0080	0.9876	0.8486
reg_lambda	0.3946	0.8350	0.8021
scale_pos_weight	7		3
**MLPClassifier**			
activation	tanh	relu	relu
alpha	0.0100	0.1000	0.0100
batch_size	15	25	5
hidden_layer_sizes	7	6	11
