# Supplementary material for: Predictors of tooth loss: A machine learning approach
Source: PLoS One. 2021 Jun 18;16(6):e0252873. doi: 10.1371/journal.pone.0252873 (PMC8213149; doi:10.1371/journal.pone.0252873)
Supplement: S5 Table — (PDF) [file pone.0252873.s008.pdf]

S5 Table: Performance of the Machine-learning Algorithms on the Test Data for Each Study Outcome with Imputed Data.

	AUC (95% CI)	ACC	Sensitivity	Specificity	F1	PPV	NPV
**Edentulism**							
Classifier							
Extreme gradient boosting trees	89.0 (87.5, 90.4)	85.1	71.8	86.0	39.3	27.1	97.7
Random forests	89.2 (87.8, 90.5)	85.0	72.9	85.9	39.7	27.2	97.8
Neural networks	87.8 (86.2, 89.2)	85.9	66.2	87.3	38.7	27.4	97.3
Light gradient boosting machine	89.3 (87.9, 90.6)	84.5	73.5	85.3	39.0	26.5	97.8
Logistic regression	87.1 (85.4, 88.6)	84.5	71.8	85.4	38.4	26.2	97.7
**Having fewer than 21 teeth**							
Classifier							
Extreme gradient boosting trees	88.9 (88.0, 89.8)	82.4	75.1	84.8	68.1	62.3	91.1
Random forests	88.2 (87.2, 89.1)	82.4	45.7	94.6	56.5	74.0	84.0
Neural networks	87.9 (86.9, 88.8)	83.0	60.1	90.7	63.9	68.2	87.2
Light gradient boosting machine	88.7 (87.7, 89.5)	78.5	85.2	76.3	66.5	54.5	93.9
Logistic regression	87.8 (86.9, 88.8)	83.1	54.2	92.7	61.5	71.2	85.9
**Missing any tooth**							
Classifier							
Extreme gradient boosting trees	83.3 (82.2, 84.3)	76.6	82.5	65.9	82.0	81.5	67.3
Random forests	83.1 (82.0, 84.2)	77.2	85.2	62.8	82.9	80.7	69.9
Neural networks	83.9 (82.8, 84.9)	77.3	85.3	62.7	82.9	80.6	70.1
Light gradient boosting machine	82.6 (81.5, 83.6)	69.3	97.3	18.2	80.3	68.4	78.5
Logistic regression	83.3 (82.2, 84.3)	76.9	83.0	65.6	82.3	81.5	68.0

Note. Test data: National Health and Nutrition Examination Survey (NHANES 2013-2014). We used multivariate imputation by chained equations (MICE) to impute missing data.
